# Evaluation of Effect of Low-Dose Methotrexate on Osseointegration of Implants: A Biomechanical Study on Dogs

**DOI:** 10.2174/1874210601812010546

**Published:** 2018-07-31

**Authors:** Mohamad Tavakoli, Jaber Yaghini, Ahmad Moghare abed, Meisam Malekzadeh, Dina Maleki

**Affiliations:** 1 Department of Periodontics, Isfahan University of Medical Sciences, Isfahan, Iran; 2 Department of Periodontics and Dental Sciences Research Center, Faculty of Dentistry, Guilan University of Medical Sciences, Rasht, Iran

**Keywords:** Methotrexate, Osseointegration, Dental implants, Rheumatoid arthritis, Bone-implant interface, Bone-implant contact

## Abstract

**Background:**

Methotrexate (MTX) is an immunosuppressive drug, widely used in inflammatory disturbances including rheumatoid arthritis. However, there is no consensus regarding the effect of MTX on implant osseointegration.

**Objective:**

The purpose of this experimental study was to investigate the effect of low dose MTX on Bone-Implant Contact (BIC) of dogs.

**Methods:**

Six mandibular premolar teeth (bilateral) of 8 mature dogs were extracted. After 3 months of healing, 6 implants (bone level, resorbable blast media surface) were inserted into the mandible of each dog (3 in each side). Dogs were randomly divided into a study group (receiving 2.5 mg/week MTX orally, 3 times per week for 4 weeks) and a control group each containing 4 dogs. In the 1^st^ week, postoperative BIC was evaluated in 4 dogs, two from each group. In the 4^th^ week, reverse torque and BIC were evaluated in the remaining 4 dogs. Data were analyzed with two-way ANOVA test for 95% confidence interval.

**Results:**

The reverse torque test of the 4^th^ week, showed a satisfying osseointegration. Histopathologic evaluation revealed that the BIC was significantly higher in the control group in comparison to the MTX group in the 1^st^ and 4^th^ week. In addition, the BIC of both groups were significantly increased in the 4^th^ week in comparison to the 1^st^ week in both groups.

**Conclusion:**

MTX has the potential to interfere with osseointegration process.

## 1 INTRODUCTION

Dental implants are the current replacement for missing teeth. Direct bone formation on the surface of implants, later termed as Osseointegration, was first introduced by the experiments conducted by Branemark *et al*. in the late 1960s. Comprehensively, osseointegration is a direct structural and functional connection between bone and the surface

 of a dental implant without any interference of other tissues which leads to the fixation of the dental implant in the alveolar bone [1, 2].

The first stage of Osseointegration which happens immediately after the implant insertion, is inflammation and extension of fibrin clot around the screw. The second stage is the attachment of osteoblasts to the surface of dental implant leading to a series of complex bone remodeling processes including osseointegration and bone resorption. The last stage is the formation of a close relation between bone and implant connected by collagenous filaments providing the long-term function of dental implant [3].

Some factors have been determined to affect osseointegration such as implant design and characteristics, surface properties, anatomic location, implant bed preparation for both a health and a morphologic (bone quality) context, surgical technique, systematic diseases, and intake of systemic medications [4-8].

Diseases such as Rheumatoid Arthritis (RA), Lichen Planus and Tardive Dyskinesia [7], and drugs such as Non-Steroid Anti-Inflammatory Drugs (NSAIDs) including COX-2 inhibitor, Cyclosporine A, Glucocorticoids, and immunosuppressive medications could interfere with osseointegration process and decrease the success rate of dental implants [9, 10].

Rheumatoid Arthritis (RA) is a chronic systemic disease, primarily of the joints, marked by inflammatory changes in the synovial membranes and articular structures, widespread fibrinoid degeneration of the collagen fibers in mesenchymal tissues, and by atrophy and rarefaction of bony structures. Methotrexate (MTX) is an immunosuppressive drug which is widely prescribed in patients with rheumatoid arthritis. It also eliminates the inflammation reaction which is the first stage of osseointegration [10-15]. However, Carvas *et al*. found no deleterious effect of MTX on osseointegration of implants inserted in the tibia of a rabbit model [16].

As there is no consensus regarding the effect of MTX on implant osseointegration, the purpose of the current study was to investigate the effect of low dose MTX therapy on the osseointegration process of a canine model. Our null hypothesis was that there were no significant differences in the osseointegration process of study and control groups [17-19].

## 2 MATERIALS AND METHODS

This study was conducted in accordance with the Animal Welfare Act and the Guide for the Care and Use of Laboratory Animals. Ethical clearance was obtained from the Ethics Committee of Isfahan University.

### 2.1 Inclusion Criteria

Young male dogs, age range of 16-20 months, 11 to 13 kilograms.

### 2.2 Exclusion Criteria

Domesticated, having rabies, uncontrollable behavior with the risk of animal injury, life-threatening situation during the study.

### 2.3 Sample Size

Eight (8) dogs, satisfying the inclusion criteria, divided into 2 groups.

### 2.4 Study Design

This was an experimental study to evaluate the effectiveness of low-dose Methotrexate on osseointegration of dental implants on dogs.

### 2.5 Presurgical Procedure

Healthy dogs without rabies were collected and received rabies vaccine 2 weeks prior to the surgery.

### 2.6 Surgery Procedure Stage 1

The administered general anesthesia was Acepromazine 1% (0.2 cc/kg), Ketamine 10% (10 mg/kg), and Atropine (0.04 mg/kg) (IV) and was maintained using Halothane. After providing general anesthesia, a full thickness flap elevated to the mandibular premolar region. Second, third and fourth premolar teeth of each side were sectioned through an atraumatic extraction procedure and excised using a peristome. 4-0 non-absorbable suture was used.

### 2.7 Surgery Procedure staged 2

Same general anesthesia protocol was performed after 3 months for the 2^nd^ surgery procedure. The night before surgery dogs received 20000 IU of Penicillin and Streptomycin (1g/10kg) to provide 4 days of antibiotic coverage; after 4 days, another antibiotic regimen administrated to maintain the coverage until the 8^th^ day. At this stage, a crestal incision was made at either side of mandibular premolar region and three identical implants with 4.1 mm diameter and 10 mm length (bone level, Resorbable Blast Media surface, DENTIS Korea) were placed straightforward, up to 1 mm in depth on the superior border of the alveolar bone, with more than 35-N insertion torque (each dog receiving 6 implants). Flaps were sutured with non-absorbable suture and the implants were maintained to heal to submerge.

### 2.8 Dogs Allocation

Following the flip of coin treatment selection, dogs were randomly allocated to the study and the control groups. In the study group, 4 dogs received Methotrexate (2.5 mg/week) orally two or three times per week for 4 weeks. In the control group, dogs received no medication. The general health of dogs was acceptable during the study and no obvious weigh loss was recognized.

### 2.9 Implant Evaluation

To evaluate osseointegration in dental implants in the study group and the control group, BIC in the 1^st^ and the 4^th^ week, and reverse torque in the 4^th^ week was measured.

In this study, a blind operator measured the reverse torque applying a 75 Ncm counterclockwise force using rachet. This technique is highly destructive and could solely be used in animal models.

Implants were excised by a trephine drill (size: 10 mm) and were maintained in 10% formalin solution. Specimens were mounted on resin blocks and sectioned (Accutom 50, Stuers, Copenhagen) to a thickness of 50 μm. Sections were stained with H&E staining technique. Stained sectioned were observed with a light microscope at 40× magnitude to measure the BIC. The BIC percentage was calculated for both sides of the implant on each of the 2 central sections obtained from each implant, using the actual BIC length as the numerator and the actual measured implant length of the implant as the denominator. Using Photoshop software version 7.0 (San Jose, CA, USA), 40× magnification images of the profile of the implant were obtained to reevaluate the BIC in samples. This profile image was then superimposed onto the surrounding bone. Two fields of bone, adjacent to the implant surface, one on each side of the implant, were acquired at 40× magnification. The implant profile was superimposed on the blank bone image of 3 locations: 150, 500, and 1,000 µm lateral to the original implant surface profiles. Then, the actual bone-implant contact (BIC %) was measured along each of the 3 superimposed implant interfaces and a mean BIC% was calculated.

### 2.10 Statistical Analysis

Appropriate descriptive statistics (including mean, standard deviation, minimum, and maximum) were computed. To analyze the data, two-way analysis of variance was performed using the *SPSS* version 11.5 (Chicago, IL, USA) with 95% confidence intervals.

## 3 RESULTS

In the present experimental study, 8 mature dogs with a mean age of 17.49 ± 1.05 months and mean weight of 12.30 ± 0.53 kg were divided into the control group and study group. BIC was measured in the 1^st^-week post-surgical on 4 dogs (2 dogs from the control group, 2 dogs from study group) and in the 4^th^ week post-surgical on the other 4 dogs.

**Chart 1:**
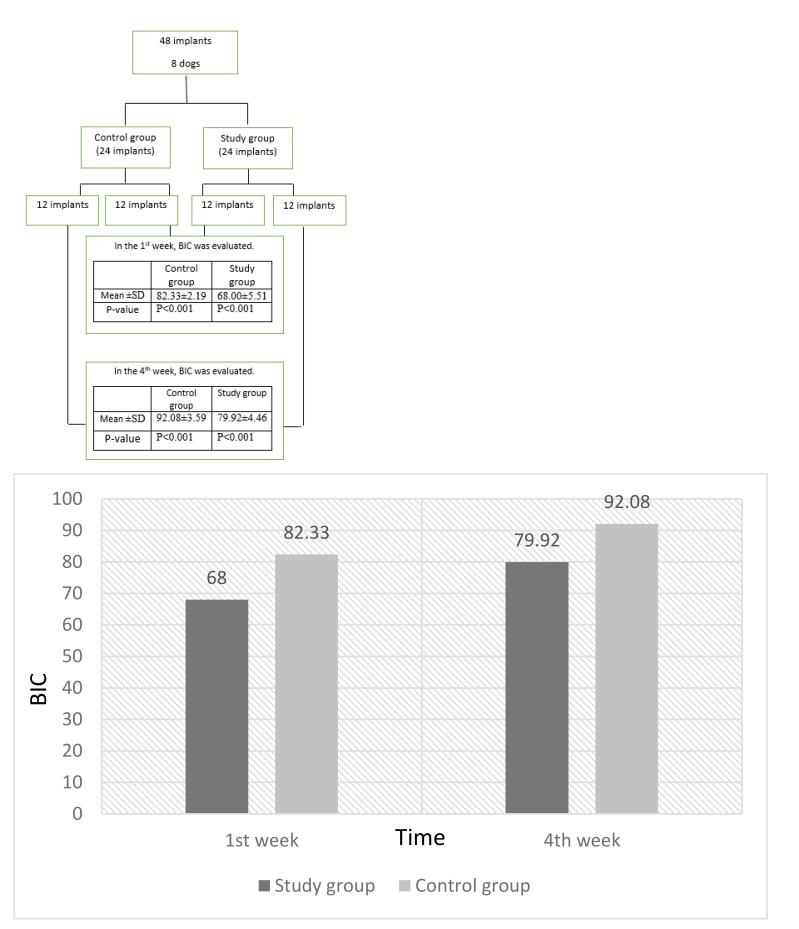
Study design and the BIC of implants according to the treatment group and time.

Two-way analysis of variance showed, the BIC was significantly increased in the 4^th^ week in comparison to the 1^st^ week in both groups. The BIC of the study group was significantly lower than the control group in both evaluation periods. In study group, BIC increased from 68 ± 5.51 at the 1^st^ week to 79.92 ± 4.64 in the 4^th^ week and in control group, it increased from 82.33 ± 2.91 in the 1^st^ week to 92.08 ± 3.59 in the 4^th^ week. (Chart **1**)

Histological evaluation showed that in study and control groups, newly formed bone was woven in the 1^st^ week and lamellar in the 4^th^ week (Fig. **1A**-**1E**).

The reverse torque of 75 Ncm was evaluated in the 4^th^ week and all of the implants in both groups of study and control showed a satisfying osseointegration with the surrounding bone.

## 4 DISCUSSION

The purpose of the current study was to evaluate the short-term effects of low-dose MTX treatment for the osseointegration of dental implants in canine models.

The most effective treatment for RA is Methotrexate (MTX), the safest Disease-Modifying Antirheumatic Drug (DMARD). Other DMARDs and biologic agents can be added to MTX in order to achieve an optimal therapeutic effect. High-dose MTX inhibits the differentiation of osteoblastic cells resulting to suppression of bone formation. However, low-dose MTX has a bone-protecting effect by suppressing osteoclasts. Inhibition of Dihydrofolate Reductase (DHFR), Thymidylate Synthase, and Aminoimidazole Carboxamide Ribonucleotide Transformylase (AICART) by MTX leads to extracellular accumulation of adenosine which inhibits T-cell activation and eliminates inflammatory reaction. As the first stage of osseointegration consists of an inflammatory reaction, MTX have the potential to interfere the osseointegration process [11-15,].

Carvas *et al*. [16] evaluated the osseointegratiom process in a rabbit model during an 18-week period. Groups of 6-8 rabbits were treated with saline, methotrexate, glucocorticoid, or methotrexate plus glucocorticoid and received implants after 6 weeks. Bone cortical thickness, total bone area around implant and BIC were measured. They found no tortious effect of MTX on the osseointegration rate which could be masked at this time. The alveolar bone of a canine model jaw has higher proximity to the humans, hence, in the present study, dogs were chosen and the BIC was evaluated in 1^st^ and 4^th^ week.

Annussek *et al*. [20] in an *in-vitro* study incubated bovine osteoblasts with various concentrations of MTX, related to tissue concentrations, over a period of fourteen days. They reported that the administration of MTX significantly reduced the proliferation of osteoblasts and mitochondrial activity. They concluded that MTX has the potential to interfere with the osseointegration process which was evident in the present study [21-27].

Suematsu *et al*. [25] studied the effect of MTX on bone turnover and reported that MTX reduces bone destruction via inhibiting RANKL receptors which play role in osteoclasts activation. Consistent with this study, Torikai *et al*. [26] found that MTX reduces the urinary concentration of resorption markers (including N-telopeptide type I collagen and deoxypyridinoline) while having no effect on the serum bone formation marker (bone alkaline phosphate) in rabbits with rheumatoid arthritis.

The difference in the results of the in-vitro studies demonstrates the possibility of osseointegration being affected by different biological mechanisms that need to be further evaluated.

The results of the current study reject the null hypothesis and reveals that BIC was increased in 1^st^ and 4^th^ week and it was higher in 4^th^ week compared to the 1^st^ week. It was significantly lower in MTX group in comparison to control group. Since the bone formed around dental implants during the 1^st^ week isn’t mature and the osseointegration is n’t complete, the microscopic findings also showed woven bone formation. However, after 4 weeks, all of the samples revealed lamellar bone around the implants which explains the increment in BIC during the 4^th^ week compared to the 1^st^ week. Although the BIC in MTX group was different to the control group in the 4^th^ week, the osseointegration was satisfying in both the groups after 4 weeks.

Further researches are needed to determine whether short term and long-term use of MTX can clinically interfere with the implant success rate in human with rheumatoid arthritis.

The effects of MTX on osseointegration was investigated in our study without the presence of inflammatory diseases *i.e*. rheumatoid arthritis which reduce the ability to adopt the results for a human model. While this can be counted as a weak point of the study design, it can also be assumed as a strong point because the changes in the osseointegration process were literally related to the use of MTX. Another limitation of this study was assessing the short-term effects of MTX, the results may be different in long-term evaluations.

## CONCLUSION

In conclusion, with limitations of the present study, low-dose MTX has the potential to interfere with the osseointegration process which could be regarded as its side effect in patients with rheumatoid arthritis in the need of implant treatment.

## Figures and Tables

**Fig. (1A):**
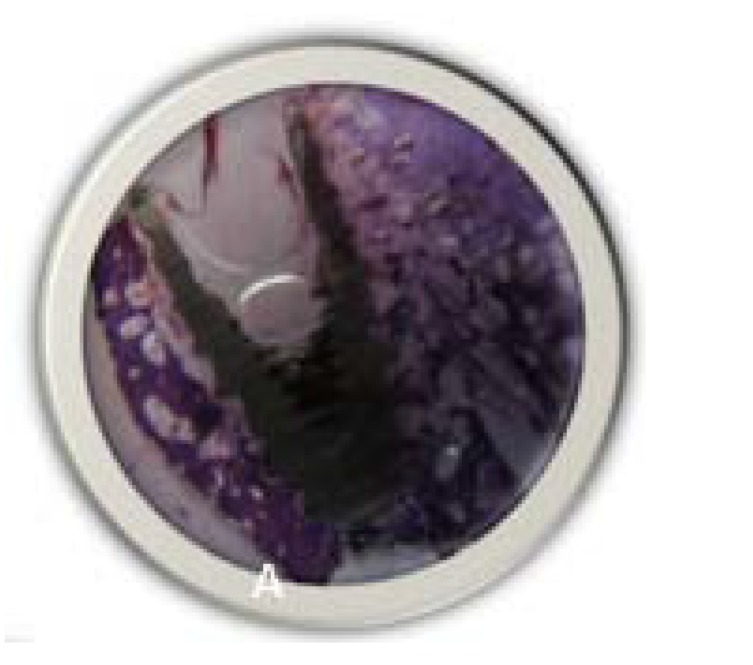
Section of the dental implant 50 μm thickness, H&E staining technique.

**Fig. (1B):**
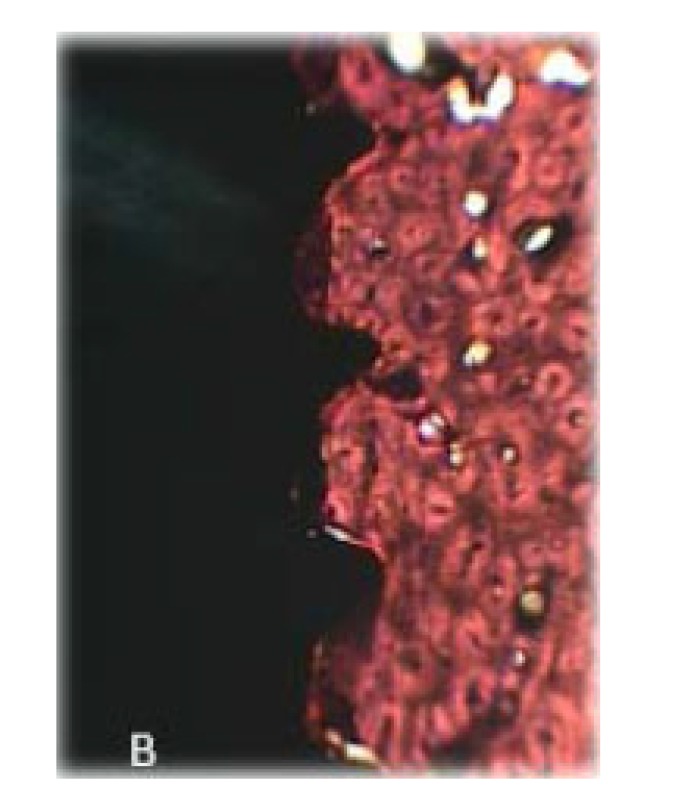
Study group in 1^st^ week.

**Fig. (1C):**
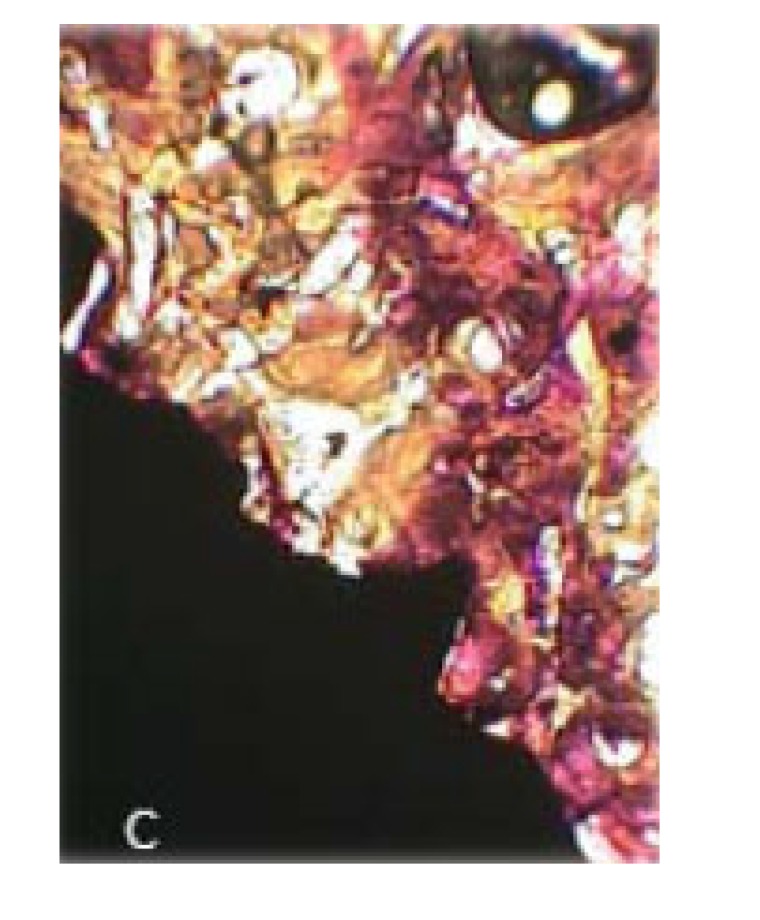
Study group in 4^th^ week.

**Fig. (1D):**
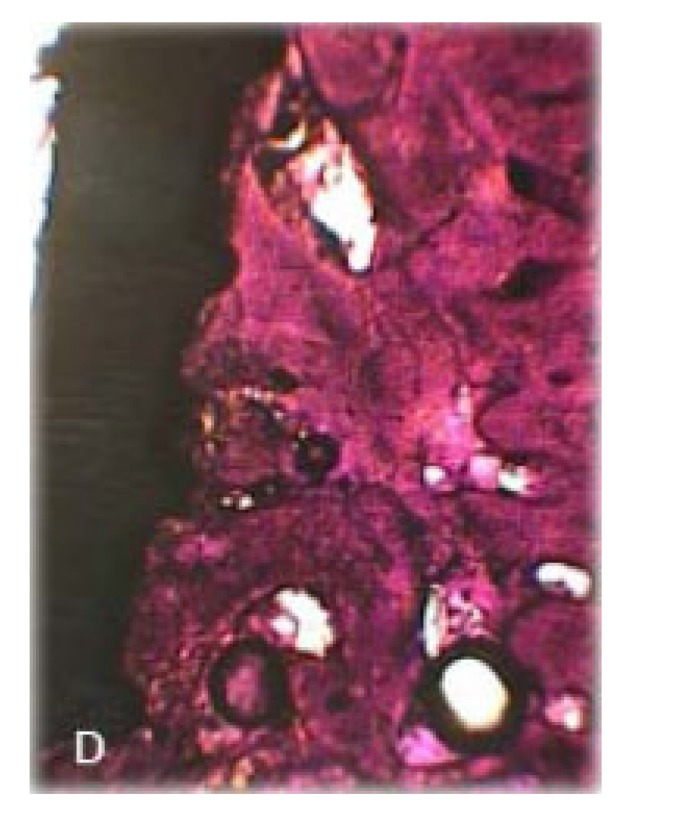
Control group in 1^st^ week.

**Fig. (1E):**
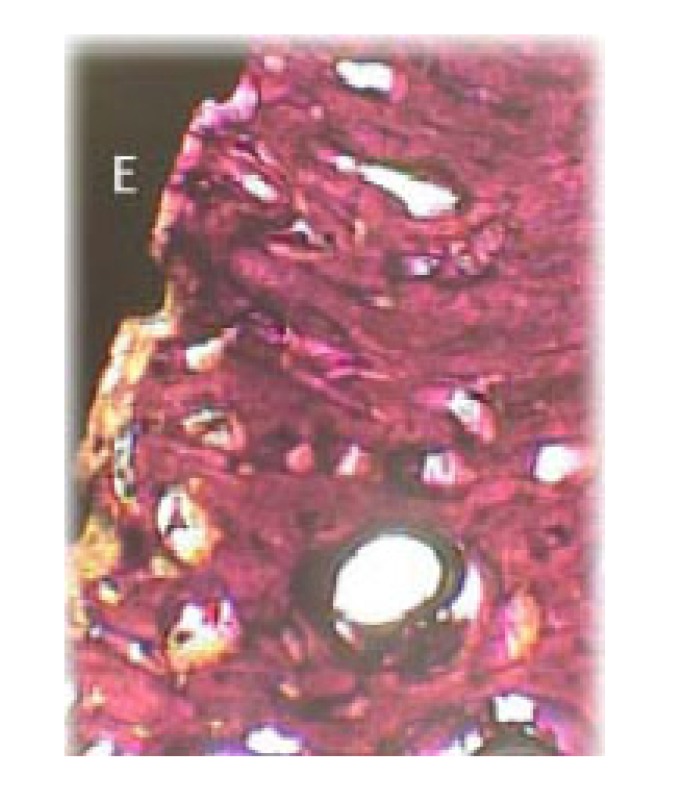
Control group in 4^th^ week.

## References

[1] Bosshardt D.D., Chappuis V., Buser D. (2017). Osseointegration of titanium, titanium alloy and zirconia dental implants: Current knowledge and open questions.. Periodontol. 2000.

[2] Trisi P., Lazzara R., Rebaudi A., Rao W., Testori T., Porter S.S. (2003). Bone-implant contact on machined and dual acid-etched surfaces after 2 months of healing in the human maxilla.. J. Periodontol..

[3] Tan N., Liu X., Cai Y., Zhang S., Jian B., Zhou Y., Xu X., Ren S., Wei H., Song Y. (2017). The influence of direct laser metal sintering implants on the early stages of osseointegration in diabetic mini-pigs.. Int. J. Nanomedicine.

[4] Yaghini J, Moghareh Abed A, Izadi M, Birang R, Torabinia N, Tavakoli M. (2017). Effect of short-term steroid use (prednisolone) on bone healing around implants: An experimental study on dogs OHDM.

[5] Parithimarkalaignan S., Padmanabhan T.V. (2013). Osseointegration: An update.. J. Indian Prosthodont. Soc..

[6] Başarir K., Erdemli B., Can A., Erdemli E., Zeyrek T. (2009). Osseointegration in arthroplasty: Can simvastatin promote bone response to implants?. Int. Orthop..

[7] Clementini M., Rossetti P.H., Penarrocha D., Micarelli C., Bonachela W.C., Canullo L. (2014). Systemic risk factors for peri-implant bone loss: A systematic review and meta-analysis.. Int. J. Oral Maxillofac. Surg..

[8] Sugerman P.B., Barber M.T. (2002). Patient selection for endosseous dental implants: Oral and systemic considerations.. Int. J. Oral Maxillofac. Implants.

[9] Pablos A.B., Ramalho S.A., König B., Furuse C., de Araújo V.C., Cury P.R. (2008). Effect of meloxicam and diclofenac sodium on peri-implant bone healing in rats.. J. Periodontol..

[10] Sakakura C.E., Marcantonio E., Wenzel A., Scaf G. (2007). Influence of cyclosporin A on quality of bone around integrated dental implants: A radiographic study in rabbits.. Clin. Oral Implants Res..

[11] McInnes I.B., Schett G. (2017). Pathogenetic insights from the treatment of rheumatoid arthritis.. Lancet.

[12] Kaltsonoudis E., Papagoras C., Droso A. (2012). Current and future role of methotrexate in the therapeutic armamentarium for rheumatoid arthritis.. Int. J. Clin. Rheumatol..

[13] May K.P., West S.G., McDermott M.T., Huffer W.E. (1994). The effect of low-dose methotrexate on bone metabolism and histomorphometry in rats.. Arthritis Rheum..

[14] El Miedany Y.M., Abubakr I.H., El Baddini M. (1998). Effect of low dose methotrexate on markers of bone metabolism in patients with rheumatoid arthritis.. J. Rheumatol..

[15] Cranney A.B., McKendry R.J., Wells G.A., Ooi D.S., Kanigsberg N.D., Kraag G.R., Smith C.D. (2001). The effect of low dose methotrexate on bone density.. J. Rheumatol..

[16] Carvas J.B., Pereira R.M.R., Bonfá E., Silveira C.A., Lima L.L., Caparbo Vde.F., Mello S.B. (2011). No deleterious effect of low dose methotrexate on titanium implant osseointegration in a rabbit model.. Clinics (São Paulo).

[17] Braun J., Rau R. (2009). An update on methotrexate.. Curr. Opin. Rheumatol..

[18] Hider S.L., Bruce I.N., Thomson W. (2007). The pharmacogenetics of methotrexate.. Rheumatology.

[19] Benedek T.G. (2010). Methotrexate: From its introduction to non-oncologic therapeutics to anti-TNF-α.. Clin. Exp. Rheumatol..

[20] Annussek T., Kleinheinz J., Thomas S., Joos U., Wermker K. (2012). Short time administration of antirheumatic drugs - methotrexate as a strong inhibitor of osteoblast’s proliferation *in vitro.*. Head Face Med..

[21] Roberts W.E., Smith R.K., Zilberman Y., Mozsary P.G., Smith R.S. (1984). Osseous adaptation to continuous loading of rigid endosseous implants.. Am. J. Orthod..

[22] Johansson C., Albrektsson T. (1987). Integration of screw implants in the rabbit: A 1-year follow-up of removal torque of titanium implants.. Int. J. Oral Maxillofac. Implants.

[23] Johansson C.B., Albrektsson T. (1991). A removal torque and histomorphometric study of commercially pure niobium and titanium implants in rabbit bone.. Clin. Oral Implants Res..

[24] Pearce A.I., Richards R.G., Milz S., Schneider E., Pearce S.G. (2007). Animal models for implant biomaterial research in bone: A review.. Eur. Cell. Mater..

[25] Suematsu A., Tajiri Y., Nakashima T., Taka J., Ochi S., Oda H., Nakamura K., Tanaka S., Takayanagi H. (2007). Scientific basis for the efficacy of combined use of antirheumatic drugs against bone destruction in rheumatoid arthritis.. Mod. Rheumatol..

[26] Torikai E., Kageyama Y., Takahashi M., Nagano A. (2006). The effect of methotrexate on bone metabolism markers in patients with rheumatoid arthritis.. Mod. Rheumatol..

[27] Uehara R., Suzuki Y., Ichikawa Y. (2001). Methotrexate (MTX) inhibits osteoblastic differentiation *in vitro*: Possible mechanism of MTX osteopathy.. J. Rheumatol..

